# Replica exchange molecular dynamics simulations reveal self-association sites in M-crystallin caused by mutations provide insights of cataract

**DOI:** 10.1038/s41598-021-02728-8

**Published:** 2021-12-02

**Authors:** Sunita Patel, Ramakrishna V. Hosur

**Affiliations:** UM-DAE Centre for Excellence in Basic Sciences, Mumbai University Campus, Vidyanagari, Mumbai, 400098 India

**Keywords:** Biophysics, Computational biology and bioinformatics, Structural biology, Diseases

## Abstract

Crystallins are ubiquitous, however, prevalence is seen in eye lens. Eye lens crystallins are long-lived and structural intactness is required for maintaining lens transparency and protein solubility. Mutations in crystallins often lead to cataract. In this study, we performed mutations at specific sites of M-crystallin, a close homologue of eye lens crystallin and studied by using replica exchange molecular dynamics simulation with generalized Born implicit solvent model. Mutations were made on the Ca^2+^ binding residues (K34D and S77D) and in the hydrophobic core (W45R) which is known to cause congenital cataract in homologous γD-crystallin. The chosen mutations caused large motion of the N-terminal Greek key, concomitantly broke the interlocking Greek keys interactions and perturbed the compact core resulting in several folded and partially unfolded states. Partially unfolded states exposed large hydrophobic patches that could act as precursors for self-aggregation. Accumulation of such aggregates is the potential cause of cataract in homologous eye lens crystallins.

## Introduction

Cataract is the leading cause of blindness that affects millions of people worldwide. It is manifested by opacity in eye lens which can happen due to mutation in the crystallins leading to congenital cataract or due to post-translational modification, oxidative stress, exposure to UV radiation, heat and specific metabolites causing age related cataract. As of 2019, WHO reported 2.2 billion people around the world to have vision impairment of which 1 billion of cases could have been prevented by medical intervention^1–3^. The dominant proteins crystallins in the eye len constitute 90% of the total soluble proteins^3^. A high concentration of soluble crystallins in the cytoplasm of the lens fiber cells provide transparency and a high refractive index to the eye-lens^4,5^. Cataract is caused due to aggregation of eye lens crystallins which causes scattering of the light and prevent it from reaching the retina^5–7^. In several studies the molecular insights of the cause of cataract is elusive.

Although, crystallins are wide spread in microbes and vertebrates, they have certain unique property which is different in these two organism types. The primitive archaeal and microbial crystallins possesses Ca^2+^ binding canonical motifs and are stabilized further by binding to Ca^2+^^8,9^ while vertebrate eye-lens crystallins impart greater stability by lowering its affinity for Ca^2+^^10,11^. The structural stability of microbial crystallins can be enhanced by mutating Ser to Arg at 5th position of the canonical motifs while eye lens γC- and γD-crystallins have natural Arg77 at that place^10–13^. This signifies importance of distinct position on the crystallin domain which is responsible for its unusual stability^10,14^. Mutation(s) in these regions can cause local and/or global change in the structure which in turn can cause aggregation of crystallins^14,15^. Similar mutation from Ser to Arg in the canonical motif even stabilizes an intrinsically disordered βγ-crystallin, Hahellin in the absence of Ca^2+^ studied by employing replica exchange molecular dynamics simulations (REMD) with generalized Born﻿ (GB) implicit solvent model^16^. REMD simulation in combination with GB model is widely used in recent days because implicit treatment of solvent reduces the system size as well as solvent viscosity significantly resulting in an increase in speed of the simulations which allows faster sampling of the probable conformations. The above combination provides an optimum balance between speed and accuracy. Recently, a number of studies have been reported where REMD with GB model is used which provide results comparable to experimental findings^16–20^. For example, in our previous work, we characterized an intrinsically disordered βγ-crystallin, Hahellin in the absence of Ca^2+^ using REMD simulation and GB implicit solvent model which yielded mixture of native-like and non-native conformations. The results compare well with the NMR findings which gave narrow dispersion of chemical shifts indicating heterogeneous mixture of conformations^20^. Therefore, combination of REMD and GB implicit solvent model is now an established technique to provide reliable information comparable to experimental findings. Nevertheless, certain GB models associated with limitations^21,22^. REMD simulation with TIP3P explicit solvent and REMD simulation with GB implicit solvent model on polyalanine peptide shows significant difference in sampling of secondary structural components. There is an increase in α-helical contain in GB REMD simulation compared to experiment and explicit solvent REMD simulation^21^. In another example, the C-terminal β-hairpin of protein G was studied with GB implicit solvent which resulted in non-native conformational states in the lowest free energy minimum while in explicit solvent simulation, native hairpin was in the lowest free energy minimum^23^. Particle mesh Ewald (PME) explicit solvent simulation and GB model can result different free energy landscape at a given temperature^24^. Despite the limitations, GB model speeds up conformational sampling 1–60 folds faster depending upon the system size with reasonable compromise on accuracy^24^.

There are three common families of crystallins in the vertebrate eye lens such as α-, β- and γ-crystallins^25^. β- and γ-crystallins form a separate superfamily by having structural similarity and evolutionary relation while α-crystallin acts as molecular chaperone. γ-crystallins are the smallest and simplest member of βγ-crystallin superfamily which exist as monomer while β-crystallins exist as oligomers. A typical βγ-crystallin domain consists of two β-sheets formed from eight β-strands arranged in two Greek key motifs. The arrangement of two Greek key motifs takes place in such a way that three of the four strands of a Greek key motif form one β-sheet and the remaining one β-strand pairs with three β-strands from other Greek key motif forming the second β-sheet^9^.

The 3D structure of an archaeal protein, M-crystallin from *Methanosarcina acetivorans* has been solved by X-ray crystallography (PDB ID: 3HZ2)^26^ as well as by NMR spectroscopy (PDB ID: 2K1W)^8^. M-crystallin consists of 84 residues and belongs to βγ-crystallin superfamily by having two Greek key motifs which fold into a single domain. The primary structure of M-crystallin shows presence of Tyr corner, Trp corner and the signature sequence Y/FxxxxF/YxG of two Greek key motifs^8,26^. M-crystallin displays striking structural similarity with human eye lens βγ-crystallins although it is a single domain protein while eye lens crystallins are double domain^8,26^. Therefore, we consider this structural homologues as a simplistic model to understand the mechanistic insights of cataract. In this study, we performed mutation of the Ca^2+^ binding residues (K34D and S77D) and a hydrophobic core residue, W45R which is known to cause congenital cataract in homologous eye len γD-crystallin^27,28^ and studied by REMD simulation using GB implicit solvation model. The study led us to understand the underlying mechanism of βγ-crystallin relation to aggregation upon mutation.

## Methods

### Starting structure

M-crystallin (PDB ID: 3HZ2) from *Mithanosarsina acetavoran* is used to build starting structures of the mutants^26^. M-crystallin is a well folded protein and retains its βγ-crystallin fold both in presence and absence of Ca^2+^^8^. Simulations were performed without Ca^2+^ to monitor the effect of mutations. Single mutant of M-crystallin was generated by mutating W45 to R45 and is abbreviated as M-crystallin-SM. Double mutant of M-crystallin was made by mutating K34D and S77D in the Ca^2+^ binding canonical motifs of M-crystallin and is abbreviated as M-crystallin-DM. In the similar way, triple mutant of M-crystallin was made by mutating K34D, W45R and S77D and is referred as M-crystallin-TM. These mutations were made using PyMOL software^29^.

### Replica exchange molecular dynamics simulation

We performed REMD simulations of M-crystallin-WT and its three mutants (M-crystallin-SM, M-crystallin-DM and M-crystallin-TM). REMD is an enhanced simulation technique as it lowers the free energy barrier at higher temperatures by sampling high energy conformations which otherwise might not be accessible at a moderate temperature. In the REMD simulation, simultaneously multiple parallel runs were started at predefined temperatures which were derived from an exponential distribution. The exchange rate were maintained constant across all the replicas by keeping relatively more temperature gap at higher temperatures and lesser gap at lower temperatures. Likewise we considered sixteen replica temperatures spanning from 280 to 340 K which are 281.85, 285.39, 288.98, 292.60, 296.28, 300.00, 303.77, 307.58, 311.44, 315.36, 319.32, 323.33, 327.39, 331.50, 335.66 and 339.88 K. We chose a moderate temperature range of 280 to 340 K because our previous study on Hahellin provided results in agreement with NMR experiments^20^. Besides, there are number of other REMD studies which use similar temperature range^30–32^. The target temperature for our analysis is 300 K as it closely matches with the physiological temperature. The temperatures range for REMD simulation were derived from an exponential distribution in such a way that the temperature of interest (300 K) lies in the intermediate region so that there will be an equal probability of exchange between the neighboring replicas on either side of the target temperature. The replica exchange probability was 60% for all the REMD simulations. The conformational coordinates were exchanged between the neighboring replicas following the Metropolis algorithm^33^. This algorithm was evaluated at every 2 ps in order to facilitate the exchange of coordinates. When the Metropolis algorithm was satisfied, the exchange attempt was considered as successful and the coordinates between the neighbouring replicas were exchanged. The velocity of each atom was then rescaled to the changed target replica temperature and in this way REMD simulations were carried out.

### Simulation details

The REMD simulations were run using AMBER 14 molecular modeling package^34^ and AMBER FF99SB force field^35,36^. We also performed classical molecular dynamics (MD) simulations on M-crystallin wild type and its mutants at 339.88 K temperature which is the highest temperature of REMD simulation employing FF14SB force field^37^ in TIP3P^38^ explicit solvent. This is one of the recent force fields where backbone and side chain modifications are introduced. This force field shows overall improvement in ϕ and ψ sampling and secondary structural content^37^. The details of the explicit MD simulation method is given in the supplementary information (SI). We performed at least 120–145 ns of simulation for each of the protein. Generalized Born Onufriev, Bashford and Case implicit solvent model (GB-OBC) with igb = 5 option was used to treat solvent^39^. The advantage of using implicit solvent is that it enhances conformational sampling faster by reducing solvent viscosity^22,24,40–42^. Certain GB implicit solvent model although associated with limitation^23^, combined use of GB implicit solvent model with REMD simulations provides results in good agreement with experimental findings ^18–20,43^. The GB solvation model includes non-polar contribution of solvation energy implicitly^39^. The total energy of the solvated molecule in GB-OBC model is given as *E*_*vac*_ + *ΔG*_*solv*_ where *E*_*vac*_ is the energy of the molecule in vacuum and *ΔG*_*solv*_ is the free energy of transferring the molecule from vacuum to solvent. *ΔG*_*solv*_ is composed of electrostatic (*ΔG*_*el*_) and non-polar part (*ΔG*_*surf*_). *ΔG*_*surf*_ is proportional to total solvent accessible surface area of the molecule with proportionality constant derived from experimental solvation energies of small non-polar molecules. In GB-OBC model the effective radius of the atoms are rescaled in such a way that for deeply buried atoms it is large while for the expose atoms it is small. Thus, it minimizes the biases towards folded structure. All the simulations were done under NVT ensembles^44^. The force-field parameters, topology and coordinates were generated using *tleap* program of AMBER^34^. The side-chains of the amino acid residues which are polar and charged were adjusted to physiological pH. Energy minimization of the starting structures were carried out for 2000 cycles of which steepest decent energy minimization was performed for the first 1000 cycles, followed by conjugate gradient minimization for next 1000 cycles to minimize any atomic overlap. The possibility of unwanted rotation at high temperature was desisted by generating chirality constraints on the minimized structure. SHAKE algorithm was used to constrain bonds stretching freedom which involves hydrogen atoms^45^. The non-bonded van der Waals potential cut-off was kept at 16 Å. Langevin thermostat was used to maintain the replica temperature by weak coupling with a collision frequency of 1 ps^−1^. Equilibration molecular dynamic run was performed on the system for 200 ps. During this the temperature of each replica was increased gradually from 0 to the corresponding target temperature. Following equilibration, REMD simulation was started for the sixteen systems with 2 fs as integration time step. The exchange attempts between the neighboring replicas were made at every 2 ps. The REMD simulation were performed using *Verlet* integration algorithm. *Multisander* program of AMBER molecular modelling package was used to run the simulations^34^. A time step of 2 ps was used to write the coordinate and output files. The trajectory corresponding to each replica temperature was filtered using *cpptraj* program and backbone Rg and C^α^ RMSD analyses were determined for all (SI Fig. S1)^34^. The trajectory corresponding to the physiological temperature of 300 K was used for the data analysis. The REMD simulations were performed in the high performance computing facility instituted at Tata Institute of Fundamental Research, Hyderabad and the National PARAM Supercomputing Facility (NPSF) of C-DAC.

### Convergence

REMD simulations were performed for M-crystallin-WT, M-crystallin-SM, M-crystallin-DM and M-crystallin-TM for a total 0.8 µs. Backbone Rg and C^α^ RMSD were monitored for all trajectories corresponding to each replica temperature which show a good convergence (SI Fig. S1). Additionally, we performed convergence measurements following Sawle and Ghosh^46^ where number of clusters and cluster entropy were estimated as a function of time to ensure adequate conformational sampling. The cluster entropy was determined using − ∑P_j_ log (P_j_), where P_j_ is the probability of jth observed cluster. The C^α^ RMSD plots of all four simulations at 300 K show a steady value after 70 ns onward till the end (200 ns). Therefore, 70–200 ns stretch from each simulation is considered as equilibrated region for further analysis (SI Fig. S2, left panel). The stretch was then divided into 20 ns overlapping time segments for which number of clusters and the distribution of cluster entropy were determined (SI Fig. S2, right panel). For clustering the structures, GROMOS clustering algorithm^47^ with a cut-off of 5 Å was used. This algorithm counts the number of neighbours under a specified cut-off and takes the structure with largest number of neighbours as cluster centre and all its neighbours as the cluster members and eliminates these structures from the pool of structures. The same procedure is repeated till the remaining structures in the pool are assigned to a cluster. The number of clusters as a function of time showed a marginal increase for M-crystallin-TM while for the rest of the simulations, it was almost constant. Further, the distribution of cluster entropy was steady for all the REMD simulations indicating convergence. Hence, the trajectories corresponding to 70–200 ns at 300 K were used for data analysis.

### Analyses

For the analysis, we monitored several conformational parameters such as radius of gyration (Rg) of backbone atoms, root mean square deviation (RMSD) of C^α^ atoms with respect to X-ray structure of M-crystallin (3HZ2), root mean square fluctuation of C^α^ atoms (RMSF), network cluster layout, native contact analysis, surface charge potential determined using APBS tool of PyMol, principal component analysis, absolute entropy calculated for all the simulations following Schlitter’s method^48^ which make use the covariance matrix of C^α^ atomic fluctuations, hydrophobic solvent accessible surface area per residue (rhSASA), ionic interaction and hydrogen bond following the criteria defined by Visual Molecular Dynamics (VMD)^49^. Native contacts were determined by an *in-house* program where C^α^–C^α^ atoms in X-ray structure of M-crystallin separated at least by 3 residues (non-local contact) come closer than 6.5 Å were considered as a reference native contacts and how many of these contacts were present in a given conformation represents the number of native contacts. The percentage of native contacts of a given cluster was computed by taking average over all cluster member’s percentage. Hydrophobic solvent accessible surface area per residue was calculated following rolling ball algorithm^50^ where solvent of 1.4 Å radius was used to probe the expose surface of a hydrophobic residue which was determined using VMD. The continuous hydrophobic patch area was calculated by selecting the connected hydrophobic residues in van der Waals representation and determining their hydrophobic solvent accessible surface area together following the same rolling ball algorithm. The ionic interactions were calculated by determining all the possible distances between the positive (NE, NH1, NH2) and negative charged (OD1 and OD2) sidechain atoms of the interacting residues and an ionic interaction was considered to be present if any one of the six distances was less than 4 Å at a given time. The standard errors in ionic and hydrogen bonding interactions were determined by dividing the equilibrium segments into intervals of 10 ns from which mean and standard deviation were computed. Trajectories visualization were done using VMD software. Data analyses were done using VMD *tcl* script, Matlab^51^ and graphs were prepared using Xmgrace^52^.

### Network cluster layout

The network cluster layout is used to visualize the major conformational clusters and the connectivity between them. The ensemble of conformations resulted from the equilibrated region of each simulation were grouped into distinct clusters based on their conformational similarity using network clustering method. The layout was generated from the pairwise C^α^ RMSD matrix of the ensemble of conformations. A pairwise cut-off value was used to establish connections between the conformations. A network cluster layout was built from nodes and links. Each node represents a conformation and the link connecting the nodes were built based on the chosen pairwise cut-off. Choosing the right cut-off is crucial because taking a larger cut-off can result a single cluster while taking a smaller cut-off can lead to fragmented clusters. So, we chose a cut-off close to the mean of pairwise C^α^ RMSD distribution as described by Ahlstrom et al.^53^. Profuse-force-directed algorithm^54^ implemented in Cytoscape version 3.5.1^55^ was used for its construction and visual representation. Nodes were assigned with similar repulsive charges while links were treated as springs of attractive forces having same spring constants. The entire system was considered as pseudo-physical system and was subjected to minimization during which more links of attractive forces can overcome the repulsive charges of nodes resulting in a well disperse network layout. In the resulted network the highly connected nodes form a single cluster while sparsely connected nodes stay apart. Average linkage algorithm implemented in MATLAB was used to generate the centroid structure of a cluster^56^.

## Results and discussion

### Choice of specific mutations in M-crystallin

**Figure 1:**
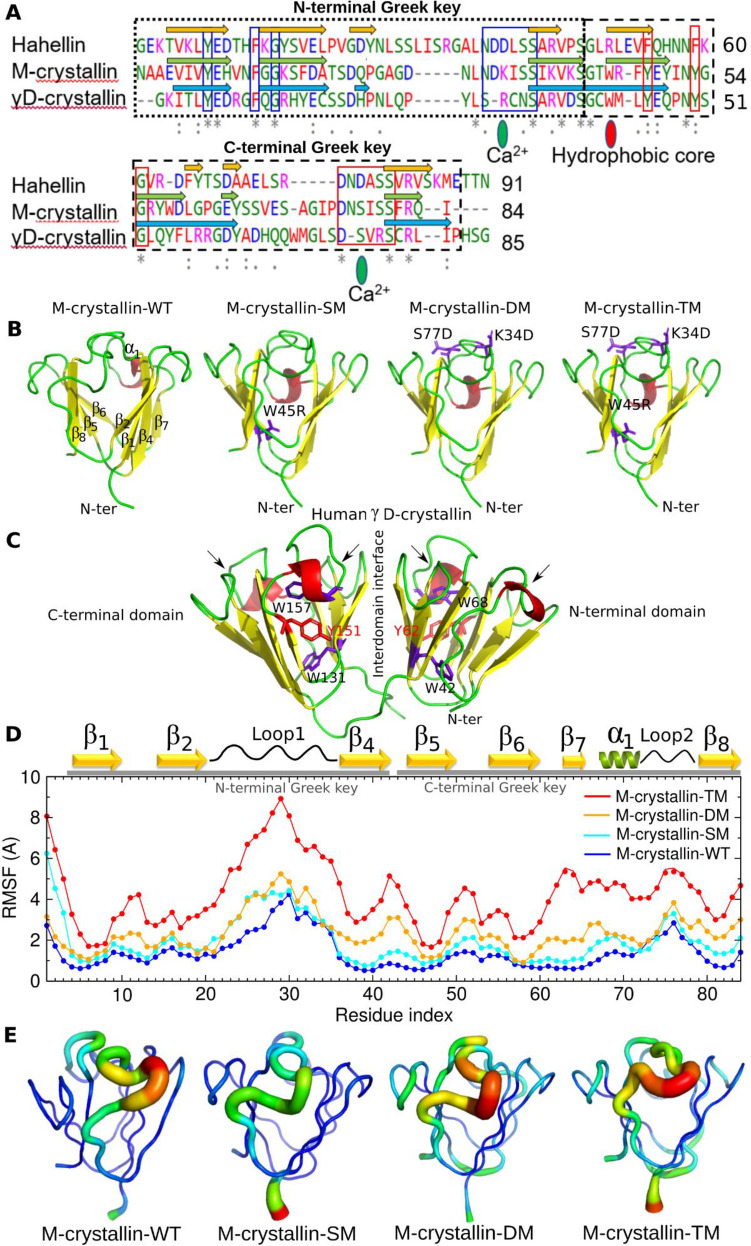
(**A**) Multiple sequence alignment of Hahellin (2KP5), M-crystallin (3HZ2) and human eye-lens γD-crystallin (1HK0) (one domain). The rectangular dashed boxes represent N-terminal Greek key (small dashed) and C-terminal Greek key (bigger dashed). The signature sequence of N-terminal Greek key is shown in blue lined boxes and C-terminal Greek key in red lined boxes. Amino acid code in blue: negatively charged residues; magenta: positively charged residues; red: hydrophobic residues; green: polar residues. The starting structures M-crystallin-WT and mutants used in the REMD simulations and explicit solvent MD simulations (**B**). Mutations are made in the hydrophobic core (W45R) and in the Ca^2+^ binding sites K34D and S77D are shown in purple stick representation. X-ray structure of γD-crystallin from human eye-lens (1HK0) displaying N- and C-terminal domains, inter-domain interface, and arrows pointing the inactive Ca^2+^ binding sites, two Tyr corners and four Trp residues (**C**). RMSF of C^α^ atoms in all four simulations (**D**). The relative flexibility are mapped onto the starting structure of each simulation and represented as sausage plots with B-factor colour codes (**E**). Narrow tubes with blue colour indicate rigid structure while wider tubes with cyan and green indicate intermediate flexibility and a further wider tubes with orange and red indicate high flexibility. PyMol software^29^ version 2.0.2 (https://pymol.org/2/) is used to prepare the 3D structures and sausage plots. Inkscape version 1.0.2-2 (https://inkscape.org/) is used to combine multiple figures. Grace software version 5.1.21 (https://plasma-gate.weizmann.ac.il/Grace/) is used for the RMSF plot.

Crystallins are well known for their unusual stability. Stability comes from specific arrangement of the double Greek key motifs. βγ-crystallins from microbial origin undergo further stabilization upon binding to Ca^2+^ while eye lens crystallins are stable without Ca^2+^. Microbial βγ-crystallins possess canonical motifs N/D-N/D-X_1_-X_2_-S/T-S to coordinate with Ca^2+^. In M-crystallin the canonical motifs are 32-NDKISS-37 and 75-DNSISS-80 in the N- and C-terminal Greek keys respectively (Fig. 1A). The X_1_ position of canonical motifs (K34 and S77) provide direct coordination sites for Ca^2+^ through its mainchain carbonyl. In order to understand the effect of mutations at X_1_ position in M-crystallin, we carried out K34D and S77D mutations (Fig. 1B). Several βγ-crystallins possess buried Trp and/or Tyr residues in the core surrounded by aromatic and hydrophobic residues as in M-crystallin (SI Fig. S3) which are conserved in many crystallins. Studies reveal that the Trp corner and Tyr corner are important for the βγ-crystallin domain stability^9^. In human γC and γD crystallins, there are four Trp residues, W42 and W68 in the N-terminal domain surrounding Y62 corner and W131 and W157 in the C-terminal domain surrounding Y151 corner are buried in the core (Fig. 1C). Further, they are surrounded by several other aromatic and hydrophobic residues in the core to provide enormous stability to the eye lens crystallins^14,57^. W42R mutation in γD crystallin causes congenital cataract^27^. Therefore, we mutate the conserved W45R in M-crystallin (W45 here corresponds W42 in γD Crystallin) to unravel the effect of mutation on βγ-crystallin stability and aggregation propensity (Fig. 1B).

### Conformational flexibility in the mutants of M-crystallin

Root mean square fluctuation measures the average fluctuations of each C^α^ atoms. Thus, it indicates flexibility of a given residue and identifies the highly flexible regions. The average RMSF for M-crystallin-WT is 1.4 Å for most of the residues except for the N- and C-terminal ends, loop1 between β_2_ and β_4_ strands and loop2 between α_1_ and β_8_ (Fig. 1D). The RMSF values are mapped onto the starting structure of M-crystallin-WT and represented as sausage plot which clearly indicates that loop1 and loop2 are highly flexible (Fig. 1E). In M-crystallin-SM the average RMSF value is 1.9 Å which is marginally higher than that of M-crystallin-WT. Sausage plot identifies similar regions as highly flexible region in M-crytallin-SM and follows almost similar trend as M-crytallin-WT. The only difference observed in M-crytallin-SM compared to M-crystallin-WT is that the N-terminal end is highly flexible (Fig. 1D,E). M-crystallin-DM shows relatively higher fluctuations compared to M-crystallin-WT and M-crystallin-SM with an average value of ~ 2.4 Å. For loop1 and loop2 the fluctuation is even higher (Fig. 1D,E). The M-crystallin-TM shows significantly large fluctuation with an average value of ~ 4.2 Å across the polypeptide chain except for the β_1_, β_5_, β_6_ and β_8_ strands. Here, the N-terminal end and the loop1 show dramatic increase in RMSF (Fig. 1D,E). Thus, overall higher flexibility is observed for loop1 and loop2 of all simulations which is remarkably large in M-crystallin-TM. RMSD, Rg and RMSF are also determined for these proteins from explicit solvent MD simulations performed at 339.88 K temperature (SI Fig. S4). Explicit solvent simulations are known to provide accurate results when performed for a longer time of microsecond to millisecond. In the present study simulations are carried out for about 120–145 ns which is considerably less in time scale required for equilibration in explicit solvent simulations. In spite of short simulations, we obtained variation in RMSF especially in the loop region which is comparable to GB-OBC implicit solvent model where M-crystallin-TM shows higher flexibility, followed by M-crystallin-SM, M-crystallin-DM and M-crystallin-WT which shows the lowest (SI Fig. S4). Similar findings are observed from eigenvector analysis which also shows highest magnitude of concerted motion (11.1) for M-crystallin-TM while lowest (2.8) for M-crystallin-WT as described in SI (Fig. S5). The absolute entropy calculated using Schlitter method^48^ are 70.6, 76.4, 80.0 and 86.3 J M^−1^ K^−1^ for M-crystallin-WT, M-crystallin-SM, M-crystallin-DM, M-crystallin-TM respectively suggesting M-crytallin-TM displays large conformational change, followed by M-crystallin-DM and then M-crystallin-SM while least conformational change is observed for M-crystallin-WT. There is a good correlation between RMSF, entropy and eigenvector analysis which unambiguously suggest that flexibility is more in mutants especially for N-terminal Greek key.

### Conformational clusters in mutants and wild type M-crystallin

**Figure 2:**
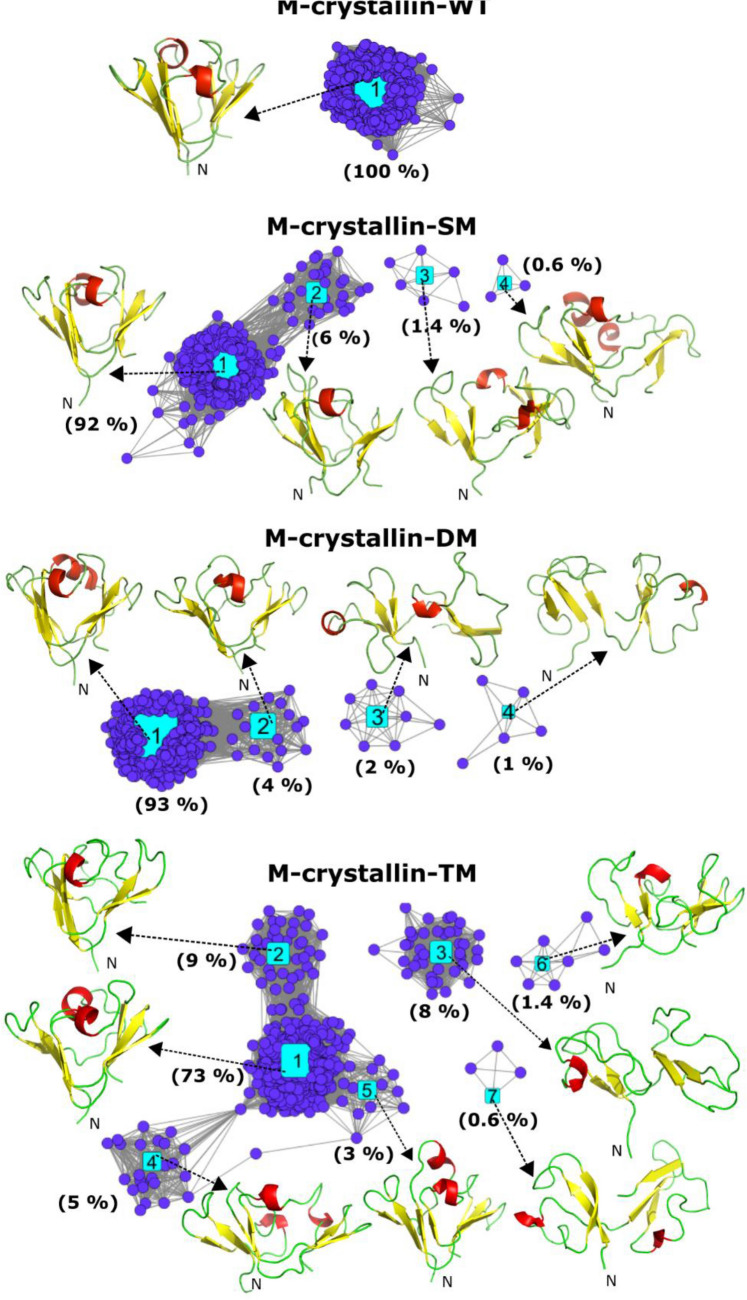
Network cluster layouts of M-crystallin wild type and its mutants. An identical pairwise C^α^ RMSD cut-off of 3.5 Å was used for constructing these network layouts. Five hundred frames were taken at a regular interval from the equilibrated region (70–200 ns) of each simulations for this analysis. A centroid structure corresponding to each cluster is pointed by an arrow. Cytoscape version 3.5.1^55^ (https://cytoscape.org/) is used to build network layout.

The C^α^ RMSD distribution determined for all the simulations illustrate that single narrow distribution for wild type while wider distributions are observed for mutants (details in SI, Fig. S6). Free energy landscape shows more numbers of distinctive minima for mutants than the M-crystallin-WT (details in SI, Fig. S7). In the network analysis, we observed single cluster accounting 100% in M-crystallin-WT. The centroid structure of the cluster is similar to the X-ray structure of M-crystallin suggesting sampling of βγ-crystallin fold (Fig. 2). In M-crystallin-SM where W45 residue is mutated to R45, the number of clusters observed are four. Out of which cluster 1 is the major cluster with 92% of population, cluster 2 is about 6% while the rest two are minor clusters having 1.4 and 0.6% of population (Fig. 2). Cluster 1 has βγ-crystallin-like fold whereas cluster 2 has deformed loops but has βγ-crystallin-like topology. The two minor clusters sample partially unfolded conformations where the central compact core is lost however βγ-crystallin-like topology is retained. In M-crystallin-DM, the network layout also shows four conformational clusters. Of which, cluster 1 is the major cluster having 93% while cluster 2 is only 4% and the rest two minor clusters are 2% and 1% of population, respectively (Fig. 2). Cluster 1 and 2 are having βγ-crystallin-like fold while cluster 3 and 4 are showing partially unfolded conformations where both Greek keys are apart. M-crystallin-TM shows seven conformational clusters. Of which, cluster 1, 2 and 5 are populated for 73, 9 and 3%, respectively. These clusters retain βγ-crystallin-like topology while cluster 3, 4, 6 and 7 are sampled for 8, 5, 1.4 and 0.6% of the population respectively are mostly in partially unfolded states (Fig. 2). Some of the conformational clusters such as cluster 3 and 4 of M-crystallin-SM look similar to cluster 4 of M-crystallin-TM while cluster 3 and 4 of M-crystallin-DM are similar to cluster 3 and 7 of M-crystallin-TM. Overall, we observed βγ-crystallin-like ensemble for M-crystallin-WT while mixture of folded and partially unfolded conformational states for mutants.

### Native contact analysis segregates conformational clusters into folded and unfolded states

**Figure 3:**
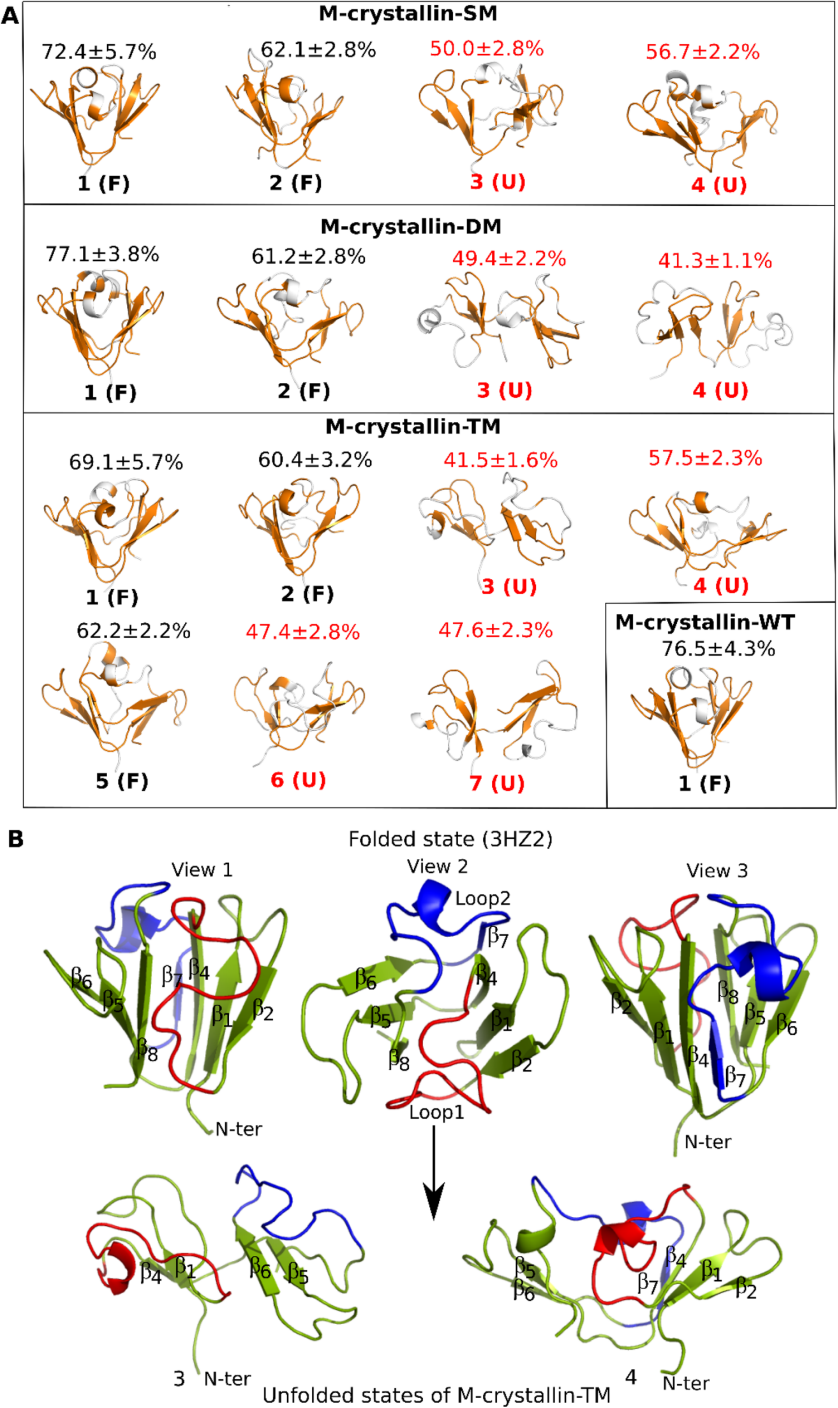
Native contact analysis was performed on the conformational clusters obtained from network cluster analysis. The average percentage of native contacts of a given cluster is indicated above the centroid structure (**A**). Clusters having 60% or greater native contacts are denoted as folded, F while the rest are designed as unfolded, U. In the centroid structures, the residues having native contacts are mapped in orange while rest of the residues are shown in white. (**B**) Three distinct views (one side view, top view, and opposite side view) of M-crystallin in X-ray structure are shown in top panel while unfolded representative states of M-crystallin-TM are shown in bottom panel. PyMol version 2.0.2 (https://pymol.org/2/) is used^29^.

Native contact percentage is used to identify whether a given cluster is folded like a βγ-crystallin or unfolded. If the percentage of native contact of a given cluster is more than 60%, it is considered as folded or else it is unfolded and are labelled as F and U respectively as shown Fig. 3A. The single cluster of M-crystallin-WT possesses 76.5 ± 4.3% of native contacts suggesting all the conformations are well folded and have βγ-crystallin-like fold (Fig. 3A). In M-crystallin-SM, out of four clusters, cluster 1 and 2 have 72.4 ± 5.7 and 62.1 ± 2.8% of native contacts and therefore are considered as folded while cluster 3 and 4 have 49.4 ± 2.2 and 41.3 ± 2.1% of native contacts thus are considered as unfolded (Fig. 3A). Similarly, in M-crystallin-DM out of four clusters cluster 1 and 2 are folded having 77.1 ± 3.8 and 61.2 ± 2.8% of native contacts while cluster 3 and 4 are unfolded having 49.4 ± 2.2 and 41.3 ± 1.1% of native contacts. In M-crystallin-TM, cluster 1, 2 and 5 possess 69.1 ± 5.7, 60.4 ± 3.2 and 62.2 ± 2.2% of native contacts respectively and therefore are folded while clusters 3, 4, 6 and 7 are having 41.5 ± 1.6, 57.5 ± 2.3, 47.4 ± 2.8 and 47.6 ± 2.3% of native contacts respectively and therefore are unfolded clusters (Fig. 3A). Native contact analysis reveals that the observed unfolded states possess ~ 40–50% of native contacts and thus are not completely unfolded like random coil but are partially unfolded states.

### Structural insights into the folded and partially unfolded states

The X-ray structure of M-crystallin has βγ-crystallin fold which has two Greek key motifs formed by two β-sheets. In this arrangement, three (β_5_, β_6_ and β_8_) of the four β-strands of C-terminal Greek key form one β-sheet and the remaining one, β_7_-strand pairs with three β-strands (β_1_, β_2_ and β_4_) of N-terminal Greek key forming the second β-sheet which are tightly linked by several interactions (Fig. 3B and SI Fig. S3). β_3_-strand is not found in M-crystallin instead a long loop1 is present in the N-terminal Greek key. There is a second loop2 in the C-terminal Greek key which extend and include unwound α-helix and β_7_-strand in the unfolded states (Fig. 3B). There are several interlocking Greek key interactions holding loop1 and β_8_-strand, and loop1 and loop2 similarly holding β_7_ and β_4_-strands together (Fig. 3B). Besides, it has several intra-β-sheet interactions in addition to the strong hydrophobic core holding the two opposite β-sheets (SI Fig. S3 and Fig. 3B). The interlocking Greek key interactions play key role for the stability of βγ-crystallin fold as unlocking these interactions can unwind the interlocked Greek keys. Although, there are several unfolded states observed in the mutants broadly they can be divided into two main types. In one type, the two interlocked Greek keys are widely separated by losing all interlocking interactions. It has only β_1_, β_4_-strands and loop1 from N-terminal Greek key and β_5_, β_6_-strands and loop2 from C-terminal Greek key (Fig. 3B, bottom left). In the second type of unfolded state, the central hydrophobic compact core is lost however it is held by loop1 and β_8_-strand, β_4_ and β_7_-strands interlocking Greek key interactions and intra-β-sheet interactions. It can be noted that M-crystallin-SM resulted in second type of while M-crystallin-DM resulted in first type and M-crystallin-TM resulted in both types of partially unfolded states.

### Destabilized interactions drive towards partially unfolded states

In order to identify the destabilizing interactions, all possible interactions are calculated. Surface charge potential is determined as shown in SI Fig. S8 which reveals that M-crystallin-WT and M-crystallin-SM display less negative surface charge potential while M-crystallin-DM and M-crystallin-TM display large negative potential on the loop regions which are important sites for Ca^2+^ binding as well as for interlocking Greek key interactions. Large repulsive interaction can break the interlocked loops from opposite Greek keys and can lead to partially unfolded states as observed in M-crystallin-DM and M-crystallin-TM. Further, total number of hydrogen bonds in each cluster are determined. However, we did not find any correlation between unfolded cluster and reduced number of hydrogen bond because certain clusters which were unfolded also have more number of hydrogen bonds than the folded one. Such situation can arise only when there are more number of non-native hydrogen bonds than the native one. Further, we determined hydrogen bonds and ionic interactions contributed by the residues undergone mutations (Table S1). Out of seven native interactions only three are observed in M-crystallin-SM, one is observed in M-crystallin-DM and none of the native interactions are observed in M-crystallin-TM suggesting non-native interactions also contribute in unfolding and drive towards partially unfolded states.

### Partially unfolded states display large hydrophobic solvent accessible surface area

**Figure 4:**
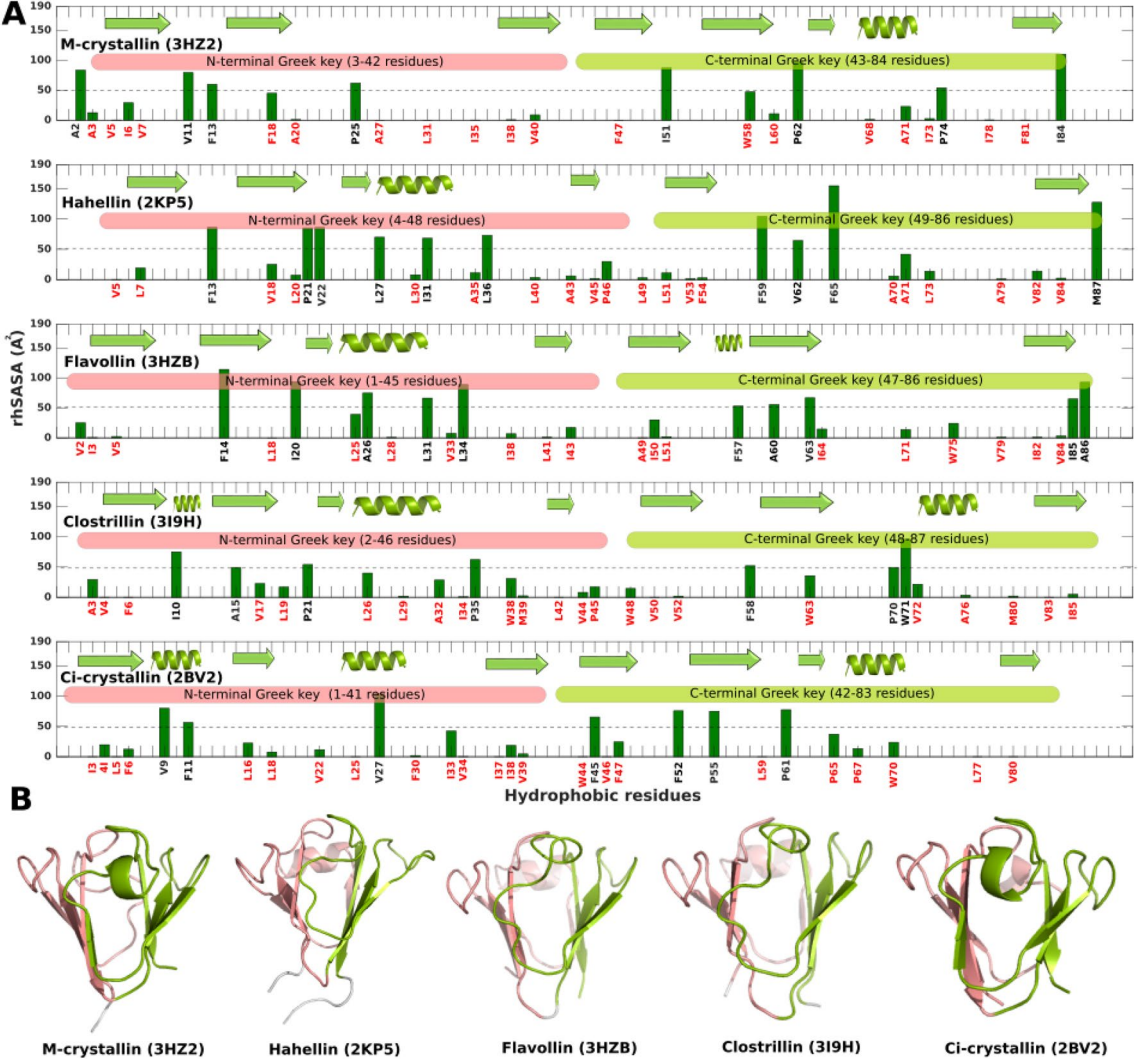
Residue specific hydrophobic solvent accessible surface area of five single domain βγ-crystallins. Residues having less than 50 Å^2^ of rhSASA are labelled in red which indicate they are buried residues. The reference rhSASA values of hydrophobic residues are Ala (102.7), Leu (184.3), Val (148.5), Ile (176.2), Phe (209.6), Pro (126.8) and Trp (251.8) Å^2^ determined from a tripeptide of the type Ala-X-Ala, where X is the residue of interest^59^ are given for relative comparison. M-crystallin (PDB ID: 3HZ2) is an archaeal protein from *Methanosarcina acetivorans*^26^, Hahellin (PDB ID: 2KP5) is a marine bacterium from *Hahella chejuensis*^60^, Flavollin (PDB ID: 3HZB) is a bacterial protein from *Flavobacterium johnsoniae*^26^, Clostrillin (PDB ID: 3I9H) is also a bacterial protein from *Clostridium beijerinckii*^26^, Ci-crystallin (PDB ID: 2BV2), is derived from tunicate *Ciona intestinalis*^61^. MATLAB R2017b (https://in.mathworks.com/) is used for the plots. PyMol version 2.0.2 (https://pymol.org/2/) is used to prepare structure^29^.

**Figure 5:**
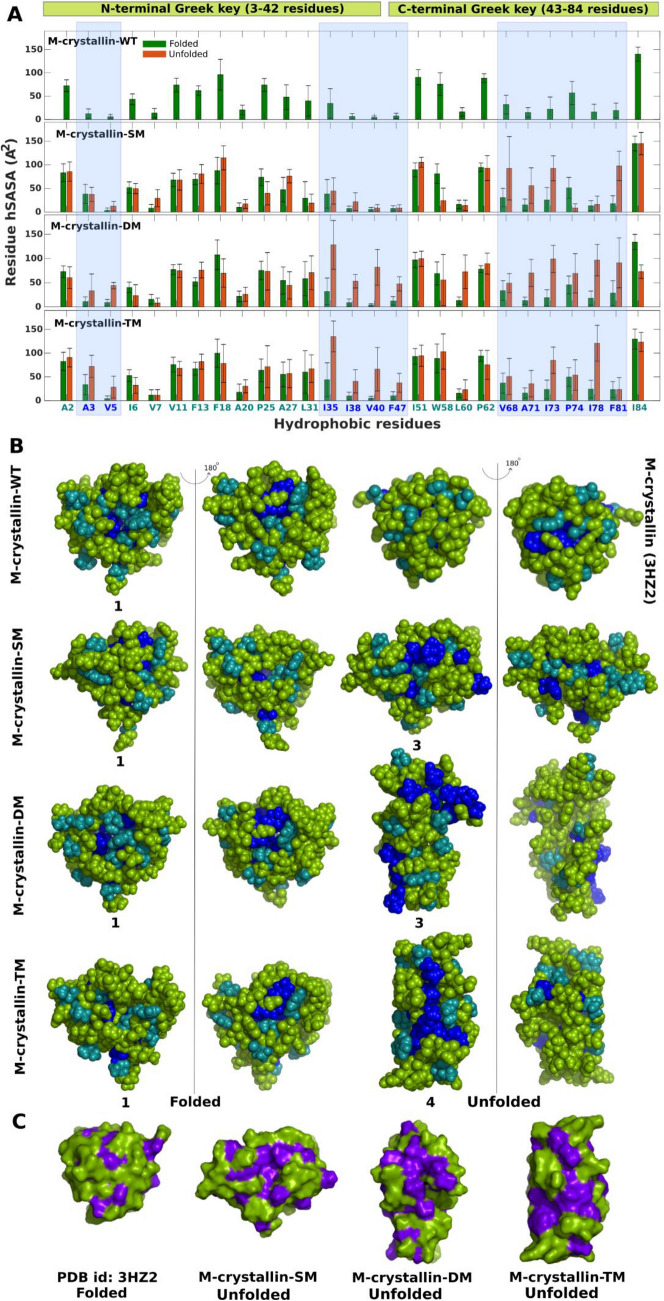
The average hydrophobic solvent accessible surface area per residue is determined for the folded or unfolded states in all simulations. For this calculation all the folded and unfolded clusters are combined and made into two separate groups. rhSASA of folded and unfolded groups are determined and are shown in bar plots (**A**). The light blue shaded boxes indicate the residues with higher hydrophobic exposure in unfolded states. These residues are mapped on the centroid structure (cluster number indicated below the centroid structure) in blue van der Waals spheres, the rest hydrophobic residues in cyan and residues other than the hydrophobic residues are in green color (**B**). Two color codes are used to indicate hydrophobes (purple) and nonhydrophobes (green) to indicate the hydrophobic patches (**C**). MATLAB R2017b (https://in.mathworks.com/) is used for the hSASA plots. PyMol version 2.0.2 (https://pymol.org/2/) is used to show hydrophobic surface area^29^.

We calculated hydrophobic solvent accessible surface area per residue in five known single domain βγ-crystallin proteins (M-crystallin, Hahellin, Flavollin, Clostrillin and Ci-crystallin) from protein data bank (Fig. 4). In spite of sequence difference, the common patterns emerged from these βγ-crystallins are that the completely buried hydrophobic residues are located at the junction of the two Greek key motifs (Fig. 4). Secondly, the hydrophobic residues located in the N-terminal Greek key motif are relatively more exposed than the C-terminal Greek key motif. These findings clearly indicates that there is a characteristic pattern of hydrophobic burial in βγ-crystallin fold which is probably necessary for the βγ-crystallin domain stability. Alteration in the characteristic pattern of rhSASA might cause unfolding of the βγ-crystallin domain. Subsequently, the rhSASA is estimated for folded (F) and unfolded (U) conformational states of a given simulation which are grouped so based on their native contact percentage (Fig. 5A). In M-crystallin-SM, the rhSASA of the residues V68, A71, I73 and F81 are relatively more exposed in unfolded state compared to its folded counterpart (Fig. 5A). These residues are located in the loop2 region of C-terminal Greek key. Similarly, in M-crystallin-DM hydrophobic exposure is observed in the similar region corresponding to the residues L60, V68, A71, I73, I78 and F81. In addition, there are two more regions corresponding to the residues I35, I38, V40 and F47 located at the junction of N- and C-terminal Greek keys and residues A3 and V5 of N-terminal Greek key are showing higher values of rhSASA in the partially unfolded states compared to that of folded state. Similarly, in M-crystallin-TM similar regions as observed in M-crystallin-DM are exposed (Fig. 5). These residues showing higher values of rhSASA in the unfolded state are mapped onto the centroid structure of folded and unfolded clusters as shown in Fig. 5B. There is a remarkable difference in the hydrophobic surface exposure of a folded and unfolded conformations. The folded conformations from wild type, mutants and X-ray structure show, a significant burial of hydrophobic residues while on the partially unfolded states there is conspicuous exposure of hydrophobic residues which form continuous hydrophobic patches of the size ~ 500 to 700 Å^2^ (Fig. 5C). These sites can act as attachment sites for self-aggregation into higher molecular weight aggregates and thus can lead to cataract in homologous eye-lens crystallins. In a recent study, γS-crystallin-G18V mutation is reported to cause cataract^58^. In that study, ANS fluorescence assay and NMR method are used to probe hydrophobic exposure of γS-crystallin-G18V mutant which shows non-specific ANS binding indicating significant hydrophobic exposure.

### Causes of conformational change in the mutants of M-crystallin

We observed a modest change in RMSF of M-crystallin-SM compared to M-crystallin-WT while M-crystallin-DM shows relatively higher fluctuations and M-crystallin-TM shows remarkably higher fluctuations (Fig. 1D and SI Fig. S4). In terms of hydrophobic surface area exposure M-crystallin-DM and M-crystallin-TM show relatively larger area than M-crystallin-SM. In M-crystallin-SM unfolding is in the central core region due to W45R mutation which makes the structure floppy. However, it does not unfold completely separating the two Greek keys apart probably due to formation of an alternate weak hydrophobic cluster which causes least exposure of hydrophobic residues. The formation hydrophobic cluster is evident from Fig. 5B and C where centroid structure of cluster 1 as well as cluster 3 of M-crystallin-SM display lesser hydrophobic surface area while majority of the hydrophobic residues are still buried in the loose core. A close look at the centroid structures of M-crystallin-SM shows βγ-crystallin-like topology for all its clusters (Fig. 2). On the other hand, M-crystallin-DM and M-crystallin-TM show relatively large exposure of hydrophobic residues as can be seen in centroid 3 (M-crystallin-DM) and centroid 4 (M-crystallin-TM) of Fig. 5B and C. In M-crystallin-DM, the cluster 1 and cluster 2 are having βγ-crystallin-like topology while cluster 3 and 4 are totally distorted structures (Fig. 2). Similar observation can also be made in M-crystallin-TM. The conformational change observed in the M-crystallin-DM is due to the repulsive interaction of the negatively charged residues, K34D and S77D which are located at the Ca^2+^ binding sites. Since, there are no neutralizing Ca^2+^ bound to these sites and double mutations with the negatively charged Asp residues intensify the repulsive interactions and could cause unfolding of M-crystallin-DM. In fact, SI Fig. S8 shows relatively large negative surface charge potential in M-crystallin-DM and M-crytallin-TM compared to M-crystallin-SM and M-crystallin-WT. Synergistically, these interactions affect the structure which is reflected in the RMSF and hydrophobic surface area exposure of the mutants. The destabilizing effect of K34D and S77D mutation can easily be verified experimentally in the future. In Hahellin presence of Asp residues at the similar position in the Ca^2+^ binding sites destabilize the entire protein^20^. That is why in the absence of Ca^2+^ Hahellin adopts intrinsically disordered states^20^. Further, addition of two positively charged Arg residues (S41R and S80R) at both the Ca^2+^ binding sites reverts the intrinsically disordered states of Hahellin to a well-folded ordered state^16^.

### M-crystallin wild type is well folded even in the absence of Ca^2+^

In M-crystallin both Ca^2+^ bound form (holoform) and unbound form (apoform) are well folded as revealed by NMR study^8^. However, they are not equally stable. Thermodynamic study of apoform and holoform of M-crystallin reveals that holoform is more stable than apoform. The unfolding temperature midpoint (T_m_) for apoform is 55 °C while for holoform it is 71 °C. The enthalpy changes at the respective T_m_ are 69 and 102.2 kcal mol^−1^ for apo and holoform respectively^8^. In our REMD simulation, M-crystallin-WT sampled homogenous single conformational form indicating stability of apoform as seen in the NMR study^8^. Similar stability of apoform is also observed in tunicate βγ-crystallin^62^, protein S^63^ where removal of Ca^2+^ has no effect in CD signature and fluorescence spectra, indicating it is a common phenomenon in the βγ-crystallins which possess high intrinsic stability. The eye-len γB-crystallin too shows a weak affinity for Ca^2+^ but are highly stable^10^. The Ca^2+^ binding affinity studied from isothermal titration calorimetry (ITC) reveals that it is low for M-crystallin with a dissociation constant (K_d_) of about 80 µM, similar to the affinity seen in the eye-lens βγ-crystallins (70–100 µM)^8^. These Ca^2+^ binding affinities are much lower than the Ca^2+^ binding affinity seen in microbial βγ-crystallins which are in the range of 10–30 µM^10^. Since, M-crystallin, protein S, eye lens crystallins, etc. possess weaker affinity for Ca^2+^ absence of Ca^2+^ does not make a large difference in the structural stability of βγ-crystallin fold.

### Aromatic residues play a key role in βγ-crystallin stability

Trp and Tyr corners play important role in providing stability to the βγ-crystallin fold. Generally, the corner residues Trp and/or Tyr are located at the beginning or end of an anti-parallel β-strand which is hydrogen bonded in case of Tyr and stabilizes the β-barrel structure of the Greek key motif^14,64^. In M-crystallin, W45 forms the Trp corner and Y65 forms the Tyr corner, and are located in the hydrophobic core surrounded by several aromatic (6-Y, 2-W and 4-F) and hydrophobic residues (SI Fig. S3)^8^. In the homologous γC (14-Y, 4-W and 4-F) and γD-crystallins (14-Y, 4-W and 6-F) there are a total of 22 and 24 aromatic residues respectively. The Tyr corners in the N- and C-terminal domains of γC and γD-crystallins are Y63 and Y151 (Fig. 1C)^14^. The four Trps residues in human γC and γD-crystallins (positions 42, 68, 131 and 157) are buried within the hydrophobic core in the interior of the protein, leading to a compact of structure. W42R mutation in human γD-crystallin is associated with congenital cataracts^27^. X-ray structure of W42R reveals that minimal changes are observed in the structure w.r.t. wild type γD-crystallin. The W42R mutant of γD-crystallin produces a small population of partially unfolded states which are in chemical exchange with folded state as revealed by NMR^27,28^. In line with this, the conserved W45R mutation in M-crystallin generates folded and partially unfolded states in our simulation. It is presumed to be driven by formation of altered hydrophobic core triggered by W45R mutation. In another multiscale atomistic simulation of γD-crystallin having W42R mutation, adopted a distinct conformation in solution where Greek key domains are more or less intact while a large perturbation is observed in the inter-domain interface region. This mutant shows a large hydrophobic exposure and can acts as primary sites for aggregation^65^. In another simulation study, W42 is mutated to polar residues such as Lys and Arg which denatures the Greek key domain as solvent enter into the core and causes hydrophobic exposure of the core residues^66^. In all these studies W42 mutation leading to perturbation of hydrophobic core is accompanied by hydrophobic exposure which is a common event irrespective of different independent studies.

### Indirect relation between highly flexible region of M-crystallin and the region of highest hydrophobic exposure

The mutants of M-crystallin show high flexibility for the N-terminal motif as evident from RMSF and eigenvector analysis. However, this highly flexible region does not correlate directly with the highest hydrophobic exposure. Most of the hydrophobic residues at the N-terminal Greek key do not show any difference in rhSASA between folded and unfolded conformations (Fig. 5) while significant difference is seen for the C-terminal Greek key as most of the hydrophobic residues in this region are exposed in partially unfolded states while they are buried in the folded state. The flexibility in the N-terminal is enhanced by breakage of several interactions connecting loop1 and β_8_-strand and loop1 and loop2 which in turn cause exposure of the C-terminal Greek key hydrophobic residues and can drive intermolecular aggregation. Recently, NMR study and dynamics light scattering (DLS) data reveal formation of oligomer in M-crystallin as a result of lowering the temperature which is accompanied by large dynamics in the Ca^2+^ binding sites^15^. There is an intricate relation between unfolding and aggregation^5,67,68^. V75D, W42R mutations in γD-crystallin^28,65,66,69^, G75V mutation in γS-crystallin^69^, S228P in βB1-crystallin^70^ are associated with partial unfolding leading to hydrophobic exposure. Our study affirm that partially unfolded species are formed due to mutations which can serve as precursors for aggregation and can lead to cataract.

## Conclusion

In our REMD simulations of M-crystallin wild type and its three mutants, the dynamic nature of loop1 of N-terminal Greek key is clearly evident. In M-crystallin-WT no dramatic conformational change is observed because of optimal packing of hydrophobic residues in the core, and presence of several interlocking Greek key interactions. On the other hand in all mutants of M-crystallin, formation of heterogeneous mixture of folded and partially unfolded states are observed. The partially unfolded states are mainly of two types. In one type, the two Greek key motifs are apart without any significant interactions between the two Greek keys while in second type, the central hydrophobic core is largely perturbed and the compact core is lost but still held by several interactions from both the Greek keys especially between loop1 and loop2 and between loop1 and β_8_-strand. In either types of unfolded states, the buried hydrophobic residues are exposed giving rise to large hydrophobic patches. These hydrophobic patches are mainly contributed by the hydrophobic residues located at the junction of both Greek keys and at the C-terminal Greek key motif. These hydrophobic patches can provide attachment sites for association into higher order molecular aggregates. Thus, hydrophobic patches on the partially unfolded crystallin are the main determinant of βγ-crystallin aggregation. Interactions restricting the loop dynamics and promoting the strength of hydrophobic core can reduce the hydrophobic exposure and thus can prevent the aggregation of crystallin which is the prime cause of cataract.

## Supplementary Information

Supplementary Information.
