# Publisher Correction: Replica exchange molecular dynamics simulations reveal self-association sites in M-crystallin caused by mutations provide insights of cataract

**DOI:** 10.1038/s41598-021-03904-6

**Published:** 2021-12-15

**Authors:** Sunita Patel, Ramakrishna V. Hosur

**Affiliations:** UM-DAE Centre for Excellence in Basic Sciences, Mumbai University Campus, Vidyanagari, Mumbai 400098 India

Correction to: Scientific Reports, 10.1038/s41598-021-02728-8, Published online 02 December 2021

The Supplementary Information published with this Article contained an error, where the layout for Table S1 was incorrect.

This error has now been corrected in the Supplementary Information file that accompanies the original Article.

