# Supplementary material for: Replica exchange molecular dynamics simulations reveal self-association sites in M-crystallin caused by mutations provide insights of cataract
Source: Sci Rep. 2021 Dec 2;11:23270. doi: 10.1038/s41598-021-02728-8 (PMC8639718; doi:10.1038/s41598-021-02728-8)
Supplement: Supplementary file 1 — Supplementary Information. [file 41598_2021_2728_MOESM1_ESM.docx]

**Supporting Information**

**Replica Exchange Molecular Dynamics Simulations Reveal Self-association Sites in M-Crystallin Caused by Mutations Provide Insights of Cataract**

Sunita Patel^1*^, R. V. Hosur^1^

^1^UM-DAE Centre for Excellence in Basic Sciences, Vidyanagari, Mumbai, India

*Corresponding address:

Dr. Sunita Patel

Address: UM-DAE Centre for Excellence in Basic Sciences, Mumbai University Campus, Mumbai 400098, India

Email: sunita.iitb@gmail.com

Tel.: 91-22-26532132

Fax: 91-22-26532134


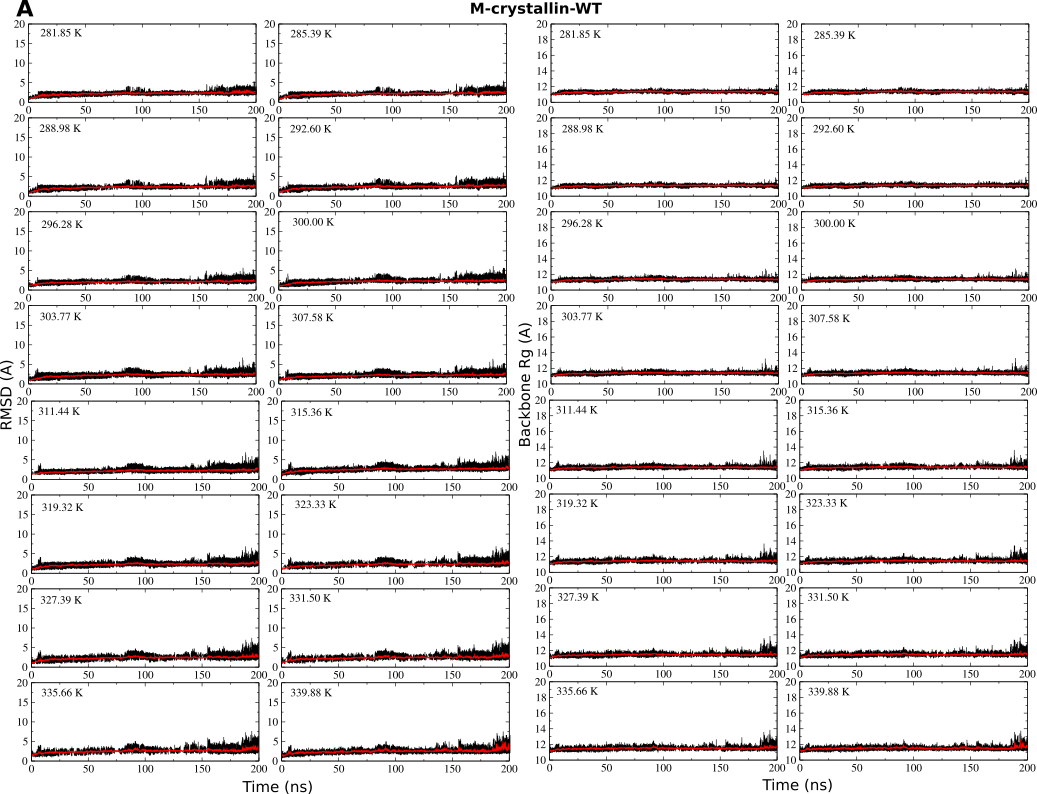


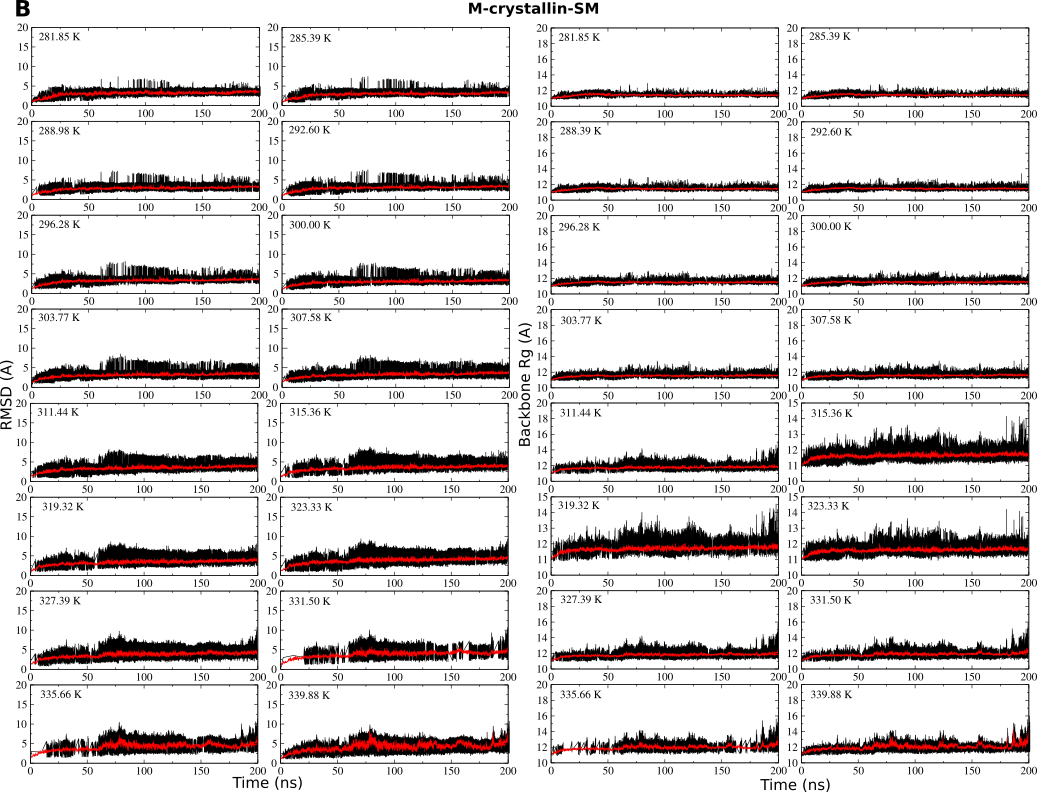


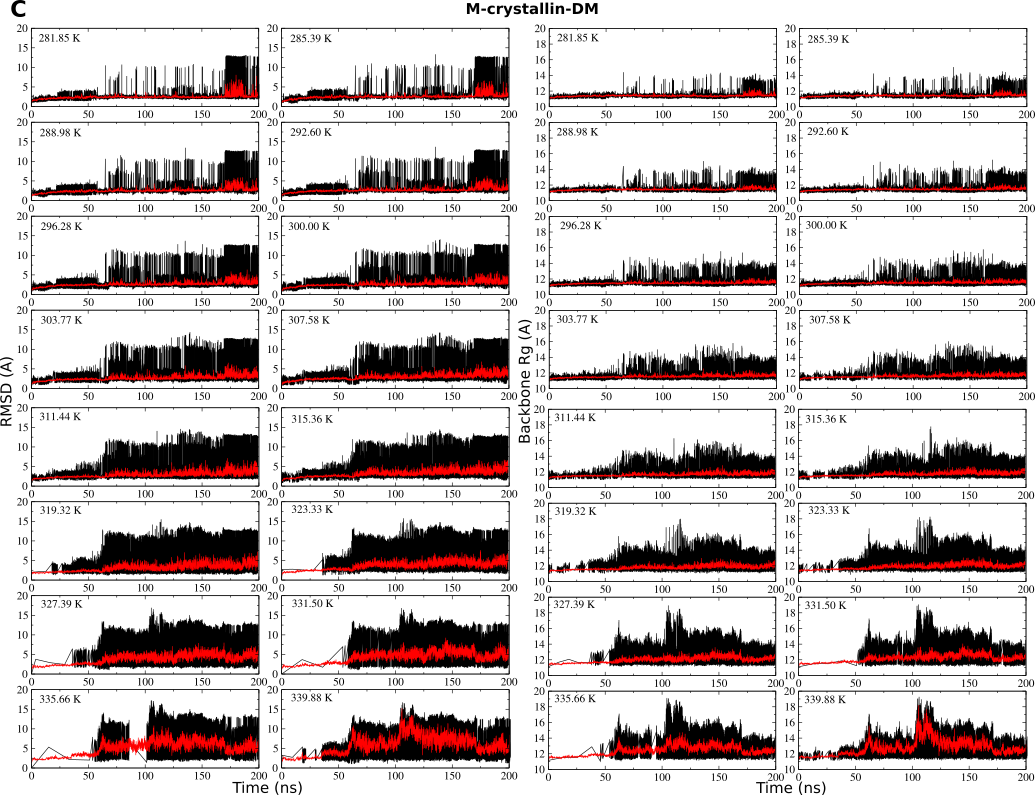


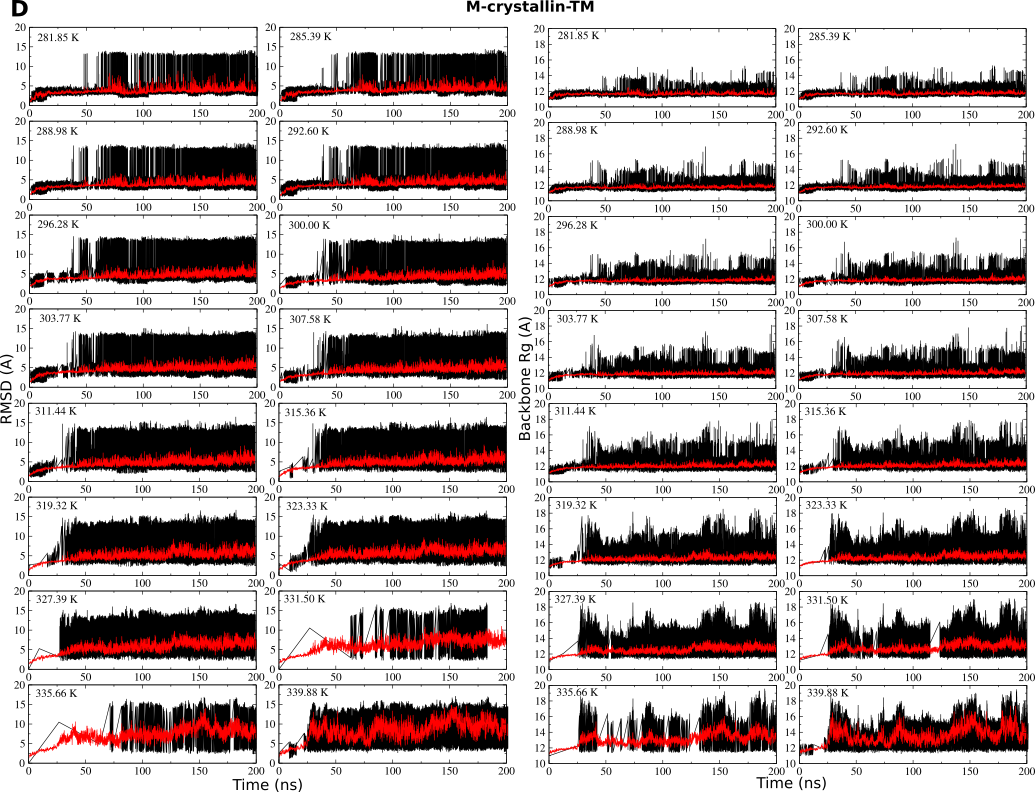


**Figure S1.** C^α^ RMSD and backbone Rg as a function of time are plotted for all replica temperature trajectories in (A) M-crystallin-WT, (B) M-crystallin-SM, (C) M-crystallin-DM and (D) M-crystallin-TM. Running averages over 50 data points are shown in red for each plot. Grace software version 5.1.21 (https://plasma-gate.weizmann.ac.il/Grace/) is used for the plots.

**
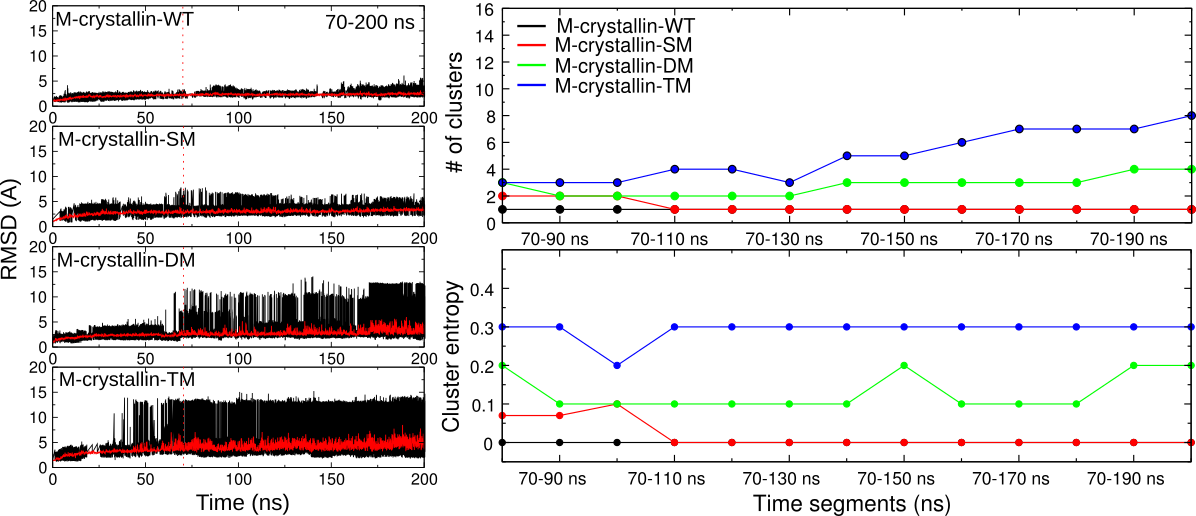
**

**Figure S2.** C^α^ RMSD (left panel) are obtained from 300 K temperature trajectories of all four simulations. Running averages over 50 data points are shown in red for each plot. Number of clusters and cluster entropy as a function of time are shown in the right panel. For clustering the structures 5Å cut off was used. Grace software version 5.1.21 (https://plasma-gate.weizmann.ac.il/Grace/) is used for the plots.

**
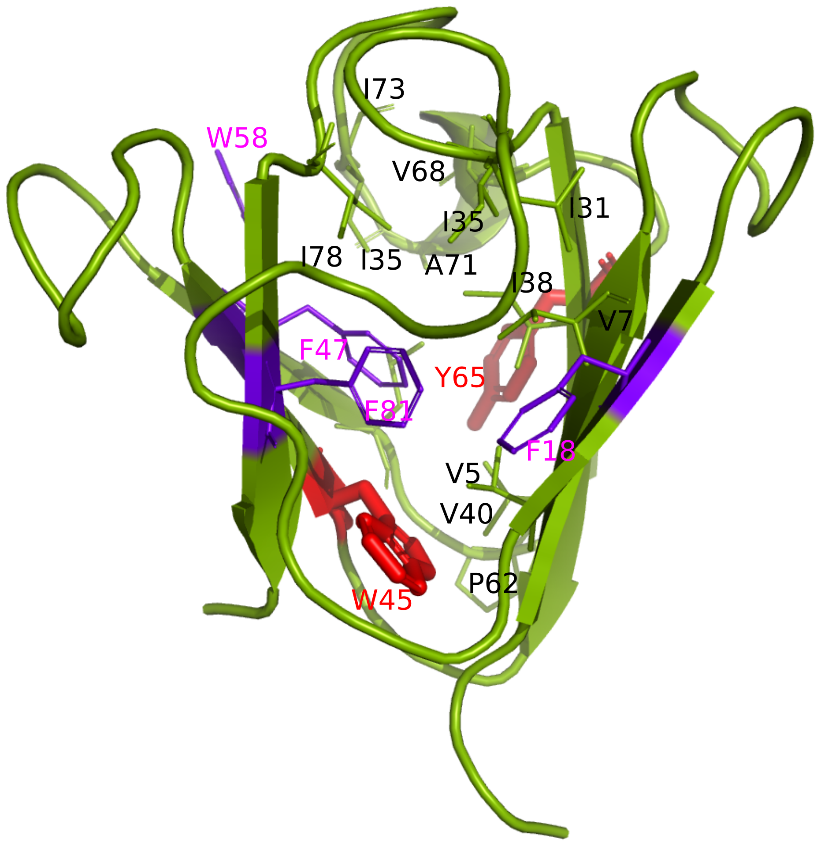
**

**Figure S3.** Trp 45 and Tyr 65 corner residues are surrounded by several aromatic (F18, F47 and F81) and hydrophobic residues in the M-crystallin X-ray structure (PDB ID: 3HZ2) forming the hydrophobic core. Corner residues are shown in red stick representation, aromatics residues are shown in purple stick and hydrophobic residues are shown in green stick. PyMol version 2.0.2 [(https://pymol.org/2/)](file:///C:\Users\aso1339\Downloads\(https:\pymol.org\2\)) is used to prepare structure^1^.

**Details of MD simulations with explicit solvent**

MD simulations on M-crystallin wild type and mutants were carried out employing FF14SB force field^2^ in TIP3P^3^ explicit solvent at 339.88 K temperature using AMBER 14 molecular modelling package^4^. This is one of the recent force fields where backbone and side chain modifications are introduced. This force field shows overall improvement in ϕ and ψ sampling and secondary structural content^2^. The topology and coordinates were generated using *tleap* program confirming to the physiological pH for amino acid side chains. Energy minimization of the protein starting structure was carried out for 2000 cycles of which first 1000 cycles was steepest decent and next 1000 cycles was conjugate gradient. TIP3P explicit solvent and counterions were added to make net charge of the system neutral. Simulations were performed under cubic periodic boundary condition where all parts of the protein are at least 10 Å away from the edge of the box. Following this a second energy minimization was carried out for the entire system. SHAKE algorithm was used to constrain all bonds involving hydrogen atoms^5^. Non-bonded van der Waals potential cut-off was set at 1 nm and electrostatic potential was calculated using particle-mesh-Ewald (PME) method with Coulomb cut-off of 1 nm and Fourier spacing of 0.12 nm and an interpolation order of 4. Temperature of the system was maintained by Langevin thermostat by weak coupling with a collision frequency of 1 ps^-1^. Position restrained molecular dynamics run was carried out under NVT condition for 200 ps and subsequently molecular dynamics equilibration without position restrain was performed for 200 ps under NPT condition. Isotropic pressure coupling was maintained by coupling to Berendsen barostat using 0.2 ps time with compressibility of 4.5e-5 bar^-1^. Subsequently, production MD run was performed with time step of 2 fs using *Verlet* integrator using *multisander* program of AMBER^4^. Coordinates were saved every 1000 steps (2 ps). We performed at least 120 to 145 ns of MD simulations for each of the protein. Simulations are performed at National PARAM Supercomputing Facility (NPSF) of C-DAC.


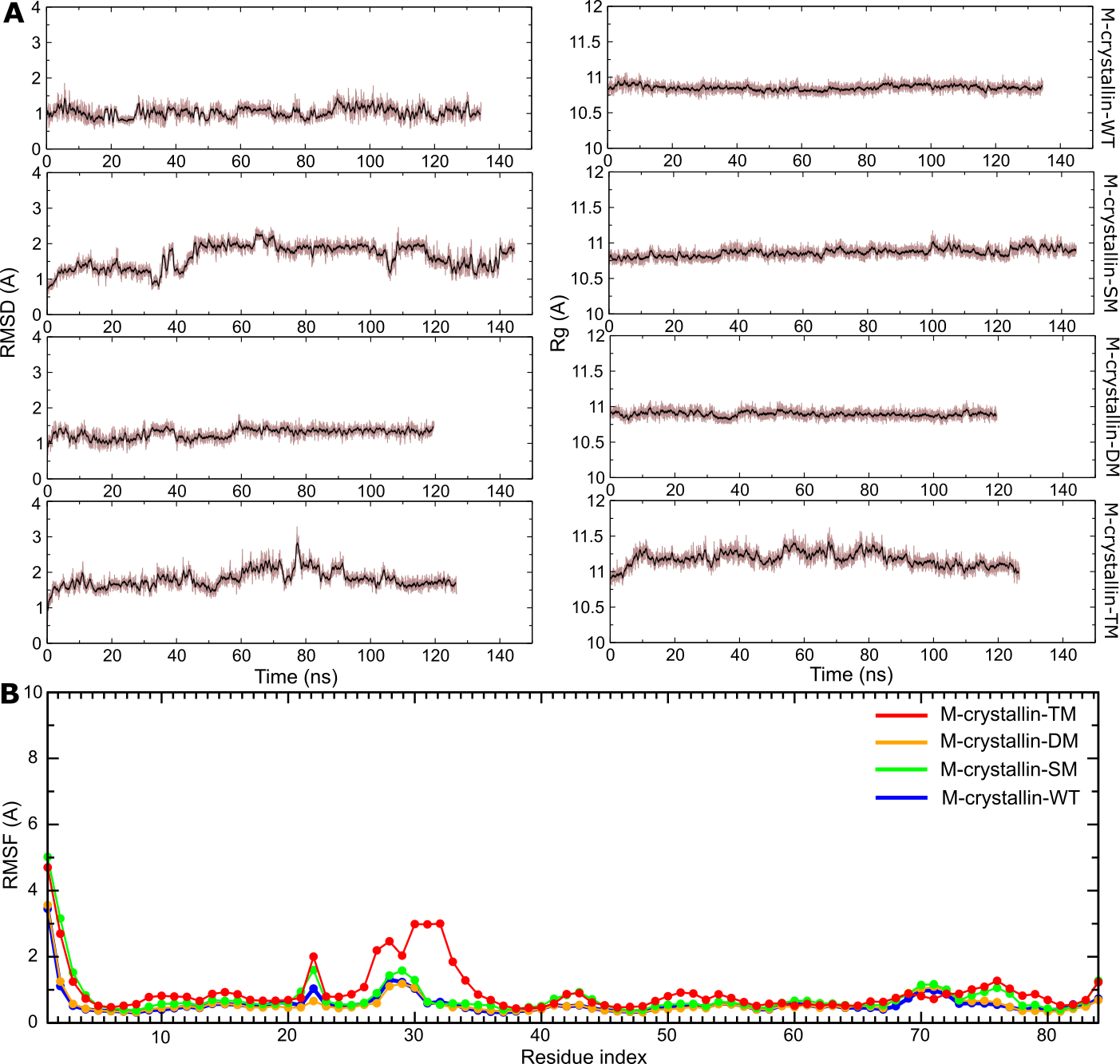


**Figure S4.** C^α^ RMSD and backbone Rg plots as a function of time determined for M-crystallin wild type and its three mutants (A) for the MD simulations performed in explicit solvent at 339.88 K. Running averages over 50 data points are shown in black for RMSD and Rg plots. RMSFs are calculated for the equilibrated region spanning from 20 ns to the end of each simulation. Grace software version 5.1.21 (https://plasma-gate.weizmann.ac.il/Grace/) is used for the plots.

**Large concerted motion observed in the N-terminal Greek key**

Protein dynamics is associated with all kind of motions in the molecule. It is possible to isolate the concerted motion from the local fluctuations by doing principal component analysis. For this purpose covariance matrix was determined for all the C^α^ atoms which was then diagonalized to determine eigenvectors and corresponding eigenvalues.

**
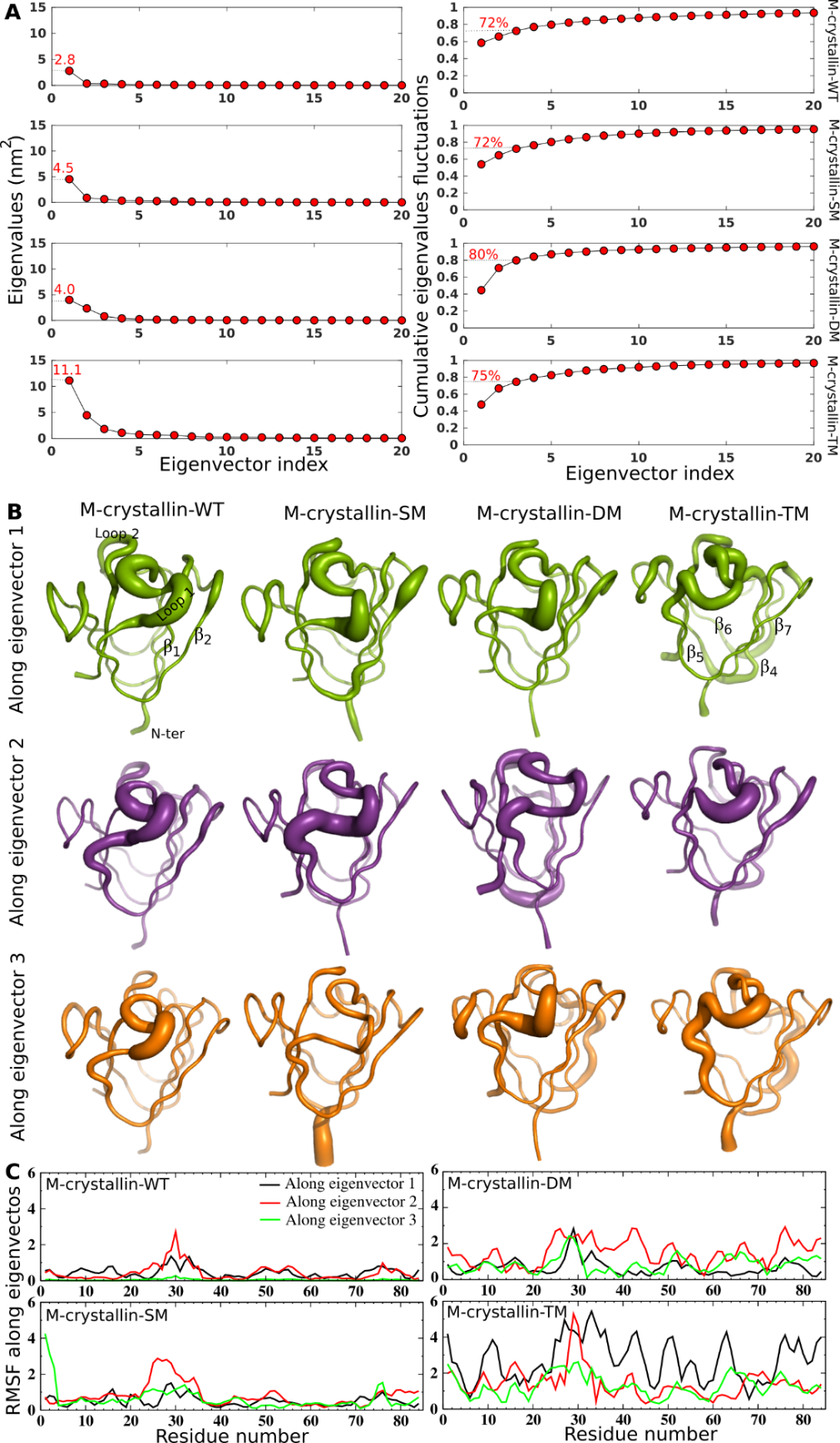
**

**Figure S5.** Eigenvectors and eigenvalues analysis on M-crystallin wild type and mutants. (A) Eigenvector index versus eigenvalues and cumulative eigenvalues functuations. (B) Sausage plots showing concerted motion along eigenvector 1, eigenvector 2 and eigenvector 3. (C) Residue number versus RMSF along eigenvector1, 2, and 3. PyMol version 2.0.2 [(https://pymol.org/2/)](file:///C:\Users\aso1339\Downloads\(https:\pymol.org\2\)) is used to prepare sausage plot^1^. Grace software version 5.1.21 (https://plasma-gate.weizmann.ac.il/Grace/) is used for the plots.

The first few eigenvectors possess higher eigenvalues and thus contribute significantly to the concerted motion. In Figure S5A, we plotted only first 20 eigenvectors and their corresponding eigenvalues as they contribute significantly to the overall dynamics. It can be observed from Figure S5A that highest magnitude of concerted motion manifested from the eigenvalue of first eigenvector in all the simulations are 11.1, 4.5, 4 and 2.8 which correspond to M-crystallin-TM, M-crystallin-SM, M-crystallin-DM and M-crystallin-WT respectively suggesting M-crystallin-TM is showing significant concerted motion. The first three eigenvectors in all the simulations contribute more than 72% of overall motional dynamics. Therefore, RMSF are determined for the projected trajectory along the eigenvector 1, 2 and 3. Flexibility are mostly observed in loop1 of N-terminal Greek key of M-crystallin-WT and M-crystallin-SM while in M-crystallin-DM and M-crystallin-TM additional regions such as loop2 and the hairpin loop between β_1_ and β_2_-strands, junction between β_4_ and β_5_-strands, between β_6_ and β_7_-strands and β_7_-strand itself show higher flexibility (Figure S4B, S4C) suggesting large concerted motion in many parts of M-crystallin-DM and M-crystallin-TM.

**Relatively increased spread of C^α^ RMSD is observed for the mutants**

The distribution of C^α^ RMSD with respect to the crystal structure of M-crystallin in wild type simulation is less than 5 Å and has only single peak at 2.2 Å suggesting sampling of homogeneous conformational forms (Figure S5). Similarly, in M-crystallin-SM mutant, the distribution of C^α^ RMSD is less than 7 Å and it shows two peaks, a major one at 2.5 Å and a minor one at 6 Å suggesting the distribution in M-crystallin-SM is broader than M-crystallin-WT. On the other hand M-crystallin-DM shows relatively larger spread of about 12.5 Å which is much higher than M-crystallin-WT and M-crystallin-SM. The C^α^ RMSD distribution shows a major peak at 2.5 Å and three minor peaks at 5, 10 and 12.5 Å suggesting sampling of heterogeneous conformations of native-like and non-native distributions for M-crystallin-DM. In M-crystallin-TM, a large variation in the C^α^ RMSD is seen which spreads up to 14 Å. The distribution shows one major peak at 3.5 Å and two minor peaks at 5.5 and 13 Å suggesting sampling of both native-like and non-native conformational forms (Figure S6). Overall, RMSD distributions suggest sampling of heterogeneous mixture of native-like and non-native conformations in contrast to single homogeneous conformation in M-crystallin-WT.


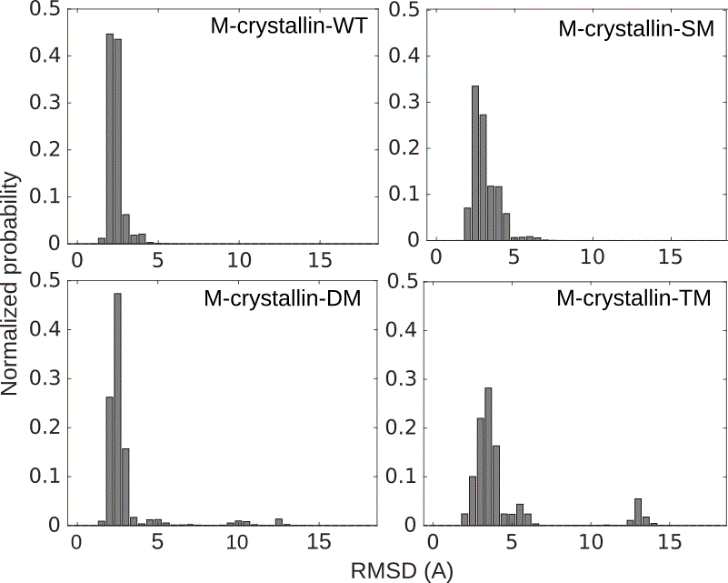


**Figure S6.** C^α^ RMSD distribution of M-crystallin-WT, M-crystallin-SM, M-crystallin-DM and M-crystallin-TM. MATLAB R2017b (https://in.mathworks.com/) is used for the plots.

**More distinct minima observed on the free energy landscape of M-crystallin mutants**

The free energy landscape was constructed using backbone Rg and C^α^ RMSD as the conformational coordinates. The Rg-RMSD plane was divided into 20 × 20 grid cells (400 cells) and free energies were calculated for each cell with respect to reference cell containing maximum number of points using the equation ΔA_ref→i_ = −RT ln(N_i_ /N_ref_), where R is the ideal gas constant and T is the temperature, N_i_ and N_ref_ are the numbers of structures in the i^th^ and reference cells, respectively. In M-crystallin-WT, there are two closely spaced minima having almost similar Rg. However, they differ in their RMSD values with one having 2.3 Å corresponding to lowest free energy minima while the other one having 4 Å is relatively higher in free energy (Figure S7). The centroid structures corresponding to these minima are structurally similar except the loop regions which differ suggesting M-crystallin-WT samples similar structures and have intact βγ-crystallin fold. In M-crystallin-SM, three minima are observed. The deepest minimum, M1, has 3 Å of RMSD and 11.4 Å of Rg, second minimum, M2, has 4.2 Å of RMSD and has 11.4 Å of Rg as that of M1 and the third minimum, M3, has 6 Å of RMSD and 12.3 Å of Rg (Figure S7). The minima M1 and M2 of M-crystallin-SM are akin to M1 and M2 of M-crystallin-WT. However, M3 of M-crystallin-SM differs largely in structure from both M1 and M2. The structure in M3 has partly unwound core and largely perturbed loops (Figure S7). The free energy landscape of M-crystallin-DM, shows five minima. The deepest minimum M1 has 2.2 Å of RMSD and 11.2 Å of Rg suggesting conformational ensemble observed in this region are well folded and have native-like conformations. The minima M2 and M5 have RMSD values of 4.7 and 6.7 Å, respectively, and have same Rg values of 12 Å. These conformational forms display similar topology however packing of the β-strands in the core is loose (Figure S7). The other two minima M3 and M4 show significantly large RMSD values of 10 and 12.5 Å and large Rg values of 13.6 and 13.3 Å, respectively, suggesting these conformations are largely unfolded and lack the typical βγ-crystallin fold (Figure S7). The free energy landscape of M-crystallin-TM also shows four minima. Out of these, M1 samples conformations similar to native-like βγ-crystallin, while M2 has partially unfolded conformation and has correspondingly higher RMSD and Rg values of 5.9 Å and 12.3 Å respectively. On the other hand M3 and M4 sample largely unfolded conformations which have higher RMSD values of 10.9 and 12.9 Å and higher Rg values of 14.9 and 13.5 Å respectively suggesting sampling of non-native conformations (Figure S7). Thus, overall, the analysis reveals that the wild type M-crystallin samples βγ-crystallin-like folded state while mutant proteins sample mixture of folded and partially unfolded conformations.


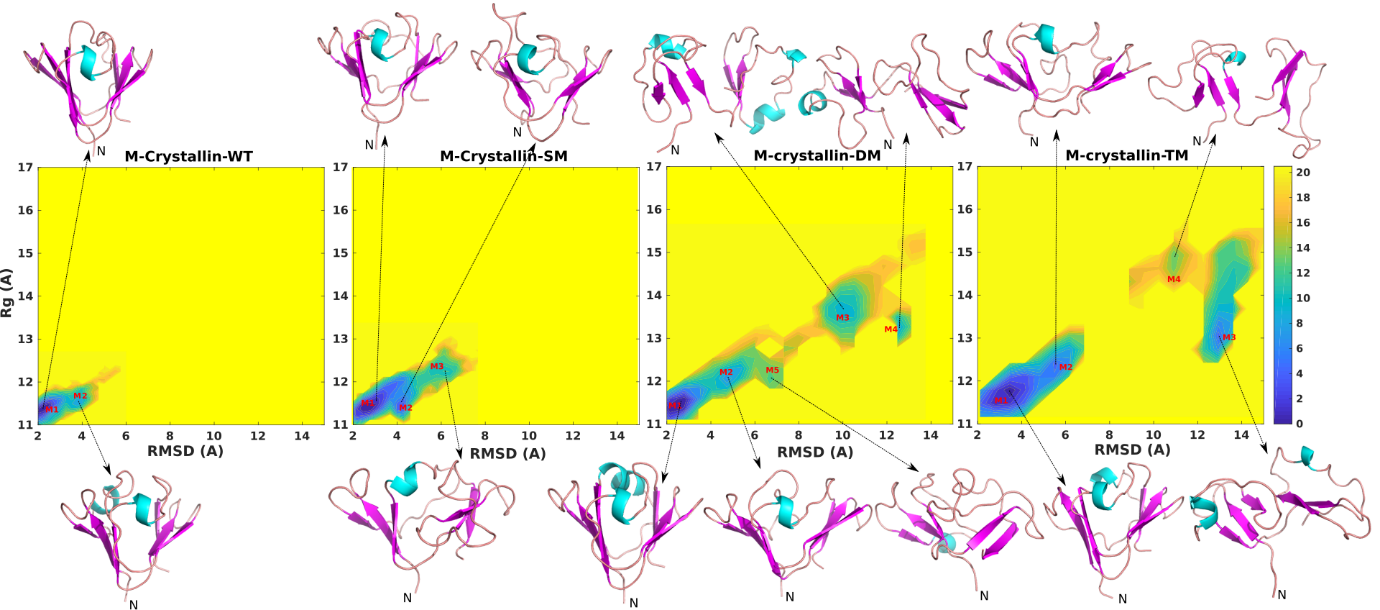


**Figure S7.** Free energy landscapes are constructed by taking backbone Rg and C^α^ RMSD as the conformational coordinates for M-crystallin wild type and its three mutants. The free energies expressed in kJ/mol are indicated on the color bar to the right. The minima on the free energy landscape are indicated by letters M1 to M5. The centroid structure corresponding to each minimum is shown by an arrow pointing to the structure. MATLAB R2017b (https://in.mathworks.com/) is used for the plots.


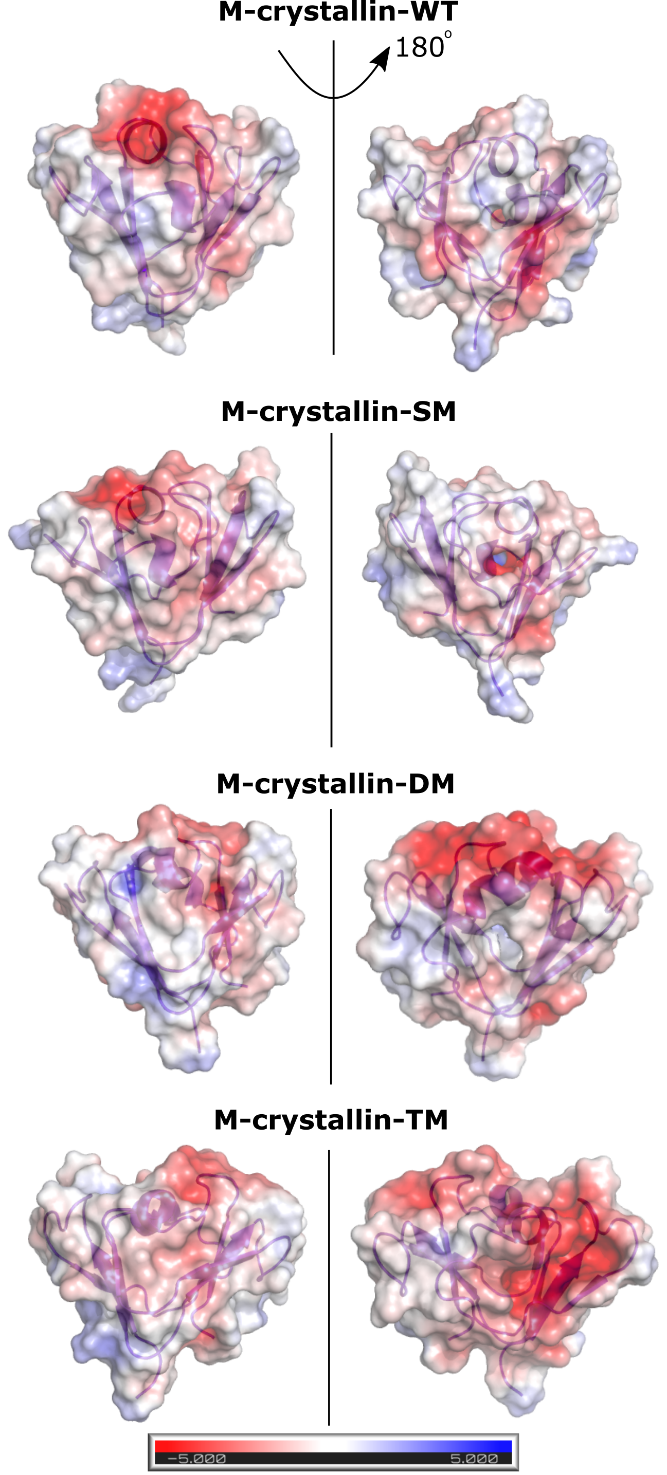


**Figure S8.** Surface charge potential shown on the centroid structure of M-crystallin-WT and the mutants. Red color indicates negative surface charge potential while blue color indicates positive charge potential while white color indicates surface with no-charge potential. PyMol version 2.0.2 [(https://pymol.org/2/)](file:///C:\Users\aso1339\Downloads\(https:\pymol.org\2\)) is used to prepare surface charge distribution^1^.

**Table S1. The hydrogen bonds and ionic interactions formed by specific residues in M-crystallin-WT which are mutated in mutant simulations. Native interactions are shown in shaded cells and ionic interactions are shown in bold.**

| **Hydrogen bond/ ionic interaction** | **Percentage (%)** | **Hydrogen bond/ ionic interaction** | **Percentage (%)** | **Hydrogen bond/ ionic interaction** | **Percentage (%)** |
| --- | --- | --- | --- | --- | --- |
| **K34 → D34** | | **W45 → R45** | | **S77 → D77** | |
|  | | **M-crystallin-WT** | |  | |
| **K34:sc-E9:sc** | 15.2±5.3 | W45:mc-L60:mc | 52.0±8.9 | S77:sc-P74:mc | 13.0±4.4 |
| **K34:sc-D29:sc** | 38.0±9.3 |  |  | S77:sc-D75:mc | 22.4±7.0 |
| K34:sc-D33:mc | 19.0±3.6 |  |  |  |  |
| **K34:sc-D75:sc** | 11.2±6.1 |  |  |  |  |
|  |  | **M-crystallin-SM** | |  |  |
| K34:mc-D29:mc | 26.6±5.2 | **R45:sc-D23:sc** | 60.2±14.7 | S77:sc-D75:mc | 23.8±4.9 |
| **K34:sc-D29:sc** | 63.2±10.9 | R45:mc-L60:mc | 52.4±11.5 |  |  |
| **K34:sc-D33:sc** | 65.0±7.9 | R45:sc-Q83:sc | 17.8±7.3 |  |  |
|  |  | **M-crystallin-DM** | |  |  |
|  |  | W45:mc-L60:mc | 52.2±10.3 | D77:sc-R56:sc | 23.4±6.1 |
|  |  | **M-crystallin-TM** | |  |  |
| D34:sc-S36:sc | 35.4±9.0 | **R45:sc-D34:sc** | 18.6±8.6 | D77:sc-R56:sc | 35.8±5.3 |
|  |  | R45:sc-L60:mc | 47.0±7.3 |  |  |
|  |  | R45:sc-Q83:sc | 16.2±4.0 |  |  |

**References**

1. DeLano, W. L. The pymol molecular graphics system, version 1.8 schrödinger, llc. Home page. http://www.pymol.org/ (accessed June 15, 2020).

2. Maier, J. A. *et al.* Ff14sb: Improving the accuracy of protein side chain and backbone parameters from ff99sb. *J. Chem. Theory. Comput.* **11**, 3696-3713, (2015).

3. Price, D. J. & Brooks, C. L., 3rd. A modified tip3p water potential for simulation with ewald summation. *J. Chem. Phys.* **121**, 10096-10103, (2004).

4. Case, D. A. *et al. Amber10, university of california,*. (2008).

5. Ryckaert, J.-P., Ciccotti, G. & Berendsen, H. J. C. Numerical integration of the cartesian equations of motion of a system with constraints: Molecular dynamics of n-alkanes. *J. Comput. Phys.* **23**, 327-341, (1977).
